# Harnessing artificial intelligence for pediatric health: Current trends and future opportunities

**DOI:** 10.1016/j.isci.2026.115962

**Published:** 2026-04-30

**Authors:** Jialin Wu, Bonan Chen, Kate Ching-Ching Chan, Mingyu Liang, Yang Lyu, Peiyao Yu, Tiejun Feng, Fuda Xie, Sifan Yu, Fengbin Zhang, Terry Cheuk-Fung Yip, Wei Kang

**Affiliations:** 1 Department of Anatomical and Cellular Pathology, State Key Laboratory of Translational Oncology, Sir Y.K. Pao Cancer Center, Prince of Wales Hospital, The Chinese University of Hong Kong, Hong Kong, China; 2 Institute of Digestive Disease, State Key Laboratory of Digestive Disease, Li Ka Shing Institute of Health Science, The Chinese University of Hong Kong, Hong Kong, China; 3 CUHK-Shenzhen Research Institute, Shenzhen, China; 4 Department of Paediatrics, Prince of Wales Hospital, The Chinese University of Hong Kong, Hong Kong SAR, China; 5 Department of Obstetrics & Gynaecology, Prince of Wales Hospital, The Chinese University of Hong Kong, Hong Kong SAR, China; 6 Law Sau Fai Institute for Advancing Translational Medicine in Bone and Joint Diseases (TMBJ), School of Chinese Medicine, Hong Kong Baptist University, Hong Kong, China; 7 Department of Gastroenterology, The Fourth Hospital of Hebei Medical University, Shijiazhuang, China; 8 Medical Data Analytics Centre, Department of Medicine and Therapeutics, The Chinese University of Hong Kong, Hong Kong, China

**Keywords:** applied sciences, artificial intelligence, artificial intelligence applications, computer science, computing methodology, health sciences, medical specialty, medicine, pediatrics

## Abstract

Artificial intelligence (AI) is transforming pediatric healthcare, offering novel opportunities for early diagnosis, personalized treatment, and more efficient clinical workflows. However, its integration into children’s health faces significant challenges due to the unique developmental, biological, and ethical considerations involved. This review explores how AI, leveraging large-scale real-world data such as electronic health records (EHRs), can augment pediatric clinical decision-making, risk stratification, communication, and workflow under human oversight. We examine its current applications and potential to improve pediatric care in areas including disease diagnosis, prediction, prevention, and personalized treatment. Additionally, we evaluate the role of AI in accelerating pediatric drug discovery and in supporting global health and epidemic management for children. Despite these promising advancements, significant barriers, such as data scarcity, ethical dilemmas, and the interpretability challenges posed by “black box” models, must be addressed to enable widespread adoption.

## Graphical abstract

**Figure:**
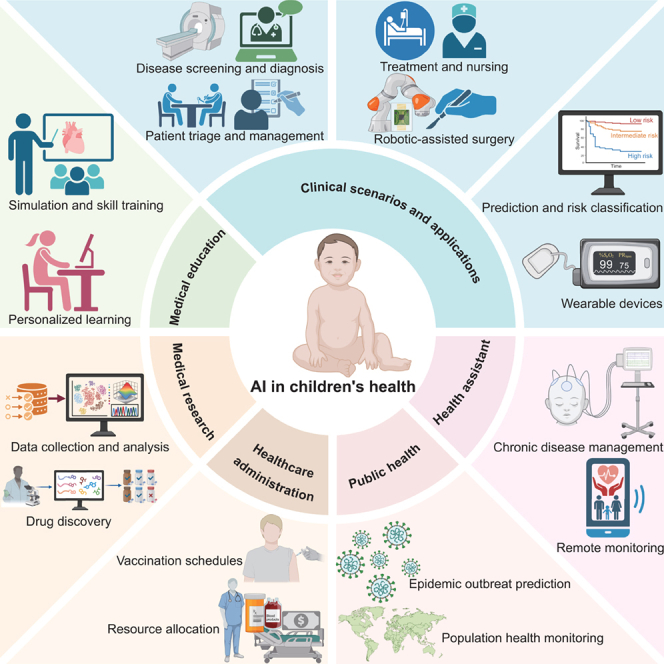

## Introduction

Artificial intelligence (AI) is reshaping modern medicine, including pediatrics.^1^^,^^2^ It brings new capacity to process complex data, uncover hidden patterns, and generate clinically useful predictions.^3^ In this broader digital transformation, pediatrics occupies a uniquely sensitive position. Children are not simply small adults; their physiological and psychological systems undergo critical developmental changes from infancy to adolescence.^4^ This developmental complexity, together with the ethical duty to protect a vulnerable population, makes pediatric care both challenging and ripe for innovation. Traditional approaches to diagnosis, treatment, and drug development have often relied on extrapolations from adult data, a practice that can miss age-specific risks and benefits.^5^

Recent advances in machine learning (ML), deep learning (DL), and large language models (LLMs) offer practical tools to augment pediatric diagnosis, prognosis, communication, and workflow, although current systems remain assistive rather than autonomous and require careful clinical oversight.^6^^,^^7^ Over the past decade, numerous prediction models have been reported to support risk stratification and personalized treatment decisions, while multimodal algorithms integrate clinical records, imaging, biosignals, and genomic data to enable earlier and more precise care.^2^ Beyond established uses, applications are expanding to patient-reported outcomes, continuous monitoring using wearables and bedside devices, and analyses that may clarify disease mechanisms. These developments hold promise for more proactive and child-centered care and may help narrow global inequities in access and quality.^8^^,^^9^ Additionally, with the rapid growth of interest in and use of LLMs in medicine, ethical principles and the need to guard against societal risks demand a nuanced understanding of LLM ethics.^10^ This review aims to provide representative coverage of recent advances in AI for pediatric health, with emphasis on pediatric-specific relevance, clinical significance, and translational potential across major application domains. It will highlight current applications and potential benefits while maintaining a critical focus on the practical hurdles and ethical considerations that must be addressed to guide future progress in this rapidly evolving field.

## AI in the core domains of pediatric health

The integration of AI in pediatric health represents a transformative leap in clinical care, diagnosis, and treatment. AI technologies, particularly ML and DL, are revolutionizing the way pediatric diseases are diagnosed and managed. In pediatric diagnostics, AI tools assist in pattern recognition and prediction, enabling more accurate and efficient identification of conditions such as neurodevelopmental disorders, congenital anomalies, and infectious diseases. Additionally, the application of DL to medical imaging has significantly reduced diagnostic errors and improved the detection of abnormalities, particularly in settings where pediatric specialists are scarce. Meanwhile, AI models may aid junior physicians in broadening their differential diagnosis, augmenting diagnostic evaluations, and supporting clinical decisions for complex or rare conditions.^11^ Beyond diagnostics, AI plays a crucial role in disease prediction and prognostication, facilitating early detection of conditions such as ophthalmic diseases, sepsis, and cancers.^12^^,^^13^ These systems analyze vast amounts of clinical data, enabling timely interventions that can substantially improve patient outcomes.^14^ Furthermore, AI’s potential in personalized medicine is being explored through the development of models that predict treatment responses and adverse events, guiding tailored therapeutic strategies for complex pediatric diseases.

Across these domains, however, the maturity of evidence remains uneven. Most pediatric AI studies are retrospective and single-center, and many emphasize discrimination metrics without sufficient reporting of calibration, subgroup performance, external validation, or prospective clinical impact. Because pediatric populations vary substantially across age, development, and care context, generalizability cannot be assumed. Accordingly, clinical readiness should be judged not only by model accuracy but also by transportability, interpretability, human oversight, and implementation feasibility. As AI continues to evolve, its ability to integrate multi-omics data across diverse ethnic populations can support the development of more generalizable models. Moreover, its potential to enhance child health across diverse settings, including low-resource environments, holds promise for bridging gaps in healthcare access and quality (Table 1).

**Table 1:** Integrative summary of major application domains of artificial intelligence in pediatric health

Domain	Predominant data type	Common AI approach	Typical validation level	Key limitations
Diagnosis and screening of current conditions	radiographs, CT, MRI, ultrasound, digital pathology, EEG, speech/video, structured EHR data, and clinical notes	convolutional neural networks, vision transformers, multimodal deep learning, NLP, classical classifiers	predominantly retrospective model development or internal validation; external validation reported in selected imaging tasks; prospective workflow evaluation remains uncommon	small pediatric datasets, variable reference standards, age-dependent phenotypes, device and site shift, limited interpretability, inconsistent subgroup reporting
Prediction, prognostication, and risk stratification	longitudinal EHR data, vital signs, laboratory results, bedside monitoring, genomics, treatment history	gradient boosting, random forest, regularized regression, survival machine learning, multimodal deep learning	mostly retrospective cohort studies; occasional temporal or external validation; prospective impact studies remain limited	calibration drift, class imbalance, dependence on local workflows, incomplete fairness analyses, uncertain clinical actionability
Prevention and public health intervention	surveillance data, immunization registries, environmental exposure data, socioeconomic indicators, healthcare utilization data	time-series forecasting, spatial/statistical learning, clustering, risk modeling	mainly observational or pilot studies with local validation; large-scale implementation studies are limited	prevention outcomes are often indirect, false positives may misallocate resources, intervention benefit is not always demonstrated, substantial context dependence, and equity concerns in population targeting
Pediatric surgery and perioperative care	perioperative EHR data, operative imaging or video, digital pathology, clinician- and family-facing text	machine-learning risk models, computer vision, NLP, LLM-assisted documentation, and communication tools	retrospective surgical cohorts and benchmark studies predominate; prospective workflow integration and safety evaluation remain limited	high-stakes safety requirements, explainability needs, hallucination risk for generative tools, medicolegal accountability, need for continuous human oversight
Drug discovery and development	multi-omics data, chemical structure libraries, HTS data, PK/PD data, real-world data, literature, and knowledge graphs	graph neural networks, representation learning, structure-based deep learning, generative chemistry, hybrid mechanistic-ML, and PBPK models	predominantly preclinical or *in silico* validation; limited pediatric clinical translation	small pediatric cohorts, developmental pharmacology and ontogeny, limited pediatric labels, long-term safety uncertainty, sparse regulatory-grade validation
Global child health and resource-constrained settings	mobile-phone images, registries, immunization data, point-of-care signals, community and geospatial data	mobile computer vision, triage algorithms, NLP-based decision support, geospatial analytics	pilot, feasibility, or local validation studies are most common; multiracial population external validation is uncommon	domain shift across settings, device variability, infrastructure and connectivity constraints, governance capacity, implementation sustainability

EHR, electronic health record; EEG, electroencephalography; HTS, high-throughput screening; NLP, natural language processing; LLM, large language model; PBPK, physiologically based pharmacokinetic; PK/PD, pharmacokinetics/pharmacodynamic; CT, computed tomography; MRI, magnetic resonance imaging.

### Diagnosis and screening of current conditions

AI has become a central tool in modern diagnostics, with wide applications in pediatric disease detection and clinical decision support.^15^^,^^16^ Within AI, ML identifies regularities in data to generate predictions, and DL is a subset that uses multilayer artificial neural networks to learn hierarchical features. Over the past decade, progress in algorithms, data availability, and computing has enabled strong performance in speech, language, and vision, and these advances are now being translated into healthcare to improve data interpretation for clinical administration, diagnosis, and outcome prediction.^5^^,^^17^ In this review, diagnosis refers to identifying a current disease state or abnormal finding, while screening refers to case-finding for current disease in asymptomatic or minimally symptomatic populations.

The Centers for Disease Control and Prevention reports that nearly 1 in 5 children experience mental, emotional, or behavioral disorders, including anxiety, depression, attention-deficit/hyperactivity disorder (ADHD), autism spectrum disorder (ASD),^18^ disruptive behavior disorder, and Tourette syndrome.^14^^,^^19^ Unfortunately, only about 20% of these children receive care from specialized psychological providers. This gap in care may be attributed to a lack of understanding of the child psychiatric workforce and the social determinants of health that make access to care more difficult for some families. Moreover, in busy clinical settings, manually reviewing large amounts of data across numerous patients to make proactive care decisions is not only impractical but also unsustainable and error-prone.^20^

Electronic health records (EHRs) provide large-scale, real-world clinical data that are increasingly used to develop clinical decision-support systems. A growing body of evidence demonstrates that ML models leveraging EHR data can continuously monitor pediatric patients and enable early identification of conditions such as sepsis,^21^^,^^22^^,^^23^ obesity,^24^^,^^25^^,^^26^ diabetes,^27^ myopia, mental health disorders,^28^^,^^29^^,^^30^^,^^31^ and hematology oncology.^32^ For instance, Su et al. developed ML models to predict suicidal behavior in 41,721 children and adolescents (ages 10–18) based on their longitudinal clinical records. The models identified both short- and long-term risk factors using deidentified, structured EHR data.^28^ Their results showed that the predictive models achieved area under the curve (AUC) values ranging from 0.81 to 0.86 across different prediction windows, with the models detecting 53–62% of suicide-positive subjects while maintaining a specificity of 90%. Although these AUC values are encouraging, they should not be interpreted as sufficient evidence of clinical usefulness on their own, because clinical usefulness depends not only on AUC or C-statistics but also on calibration, validation setting, subgroup performance, clinically meaningful decision thresholds, and whether the model improves care relative to existing practice. Beyond this, EHR-integrated ML approaches have also shown promise in the early detection of critical deterioration events,^33^ infections,^34^ rare genetic diseases,^35^ hand-foot-and-mouth disease,^36^ and in myopia prevention^37^ (Table 2). Collectively, these studies highlight the potential of ML-enabled analysis of EHR data to translate the advantages of big-data research into clinical practice, supporting precision interventions and informing pediatric health policy.

**Table 2:** Summary of machine learning models applied to electronic health record data in selected pediatric healthcare studies

Study (Year) [ref.]	Disease	Study aim	Patients	Algorithms	Main results
Angell et al.^30^	ASD	to develop ML-based models for predicting ASD diagnosis	70,803 ASD; 212,409 non-ASD children	logistic regression, XGBoost	the study identified significant fairness and bias issues in ASD prediction models based on EHR data
Rahman et al.^31^	ASD	to evaluate ML models for early ASD prediction using electronic medical records	1,397 ASD and 94,741 non-ASD children	logistic regression, neural networks, and random forest	models achieved a mean C-statistic of 0.709 with high specificity (98.2%) but limited sensitivity (29.9%)
Leroy et al.^18^	ASD	to automatically extract DSM diagnostic behaviors from EHRs using NLP	4,491 children aged 4 and 8 years	rule-based NLP parser, decision tree	the NLP parser achieved 76% precision, 43% recall, and >99% specificity, outperforming the ML baseline
Chappell et al.^32^	Acute myeloid leukemia	to evaluate ML models for predicting bloodstream infections	95 pediatric patients (median age 9.2 years)	regularized logistic regression, KNN, random forest	regularized logistic regression performed best with an AUC of 0.748 and substantially improved specificity over standard clinical criteria
Kurowski et al.^21^	Cerebral palsy	to characterize patterns of care among children with cerebral palsy in a tertiary healthcare system	6,369 children aged 0–21 years	hierarchical clustering	seven distinct care clusters were identified, including musculoskeletal/functional, neurological, urgent care, procedures, comorbidities, developmental/behavioral, and primary care
Pang et al.^24^	Childhood obesity	to predict obesity risk between ages >2 and ≤7 years	860,510 children aged 2–7 years	decision tree, Naive Bayes variants, logistic regression, neural network, SVM (RBF), XGBoost	XGBoost performed best with an AUC of 0.81, significantly outperforming other models across precision, F1 score, accuracy, and specificity at 80% sensitivity
Hammond et al.^25^	Childhood obesity	to predict obesity at age five using data from the first two years of life	52,945 children (<24 months)	L1-regularized logistic regression, random forest, gradient boosting	models predicted obesity at age five with reasonable accuracy, demonstrating the utility of early-life data without additional data collection
Dugan et al.^26^	Childhood obesity	to predict future obesity using clinical data collected before age two	7,519 children aged 2–10 years	RandomTree, RandomForest, J48, ID3, Naive Bayes, Bayes	The ID3 model showed the best performance with 85% accuracy, 89% sensitivity, 84% PPV, and 88% NPV
Shi et al.^33^	Critical deterioration	to develop machine learning models for predicting critical deterioration events	57,233 inpatients	GLM, XGBoost, deep neural network	XGBoost achieved the highest performance with a C-statistic of 0.951 (95% CI: 0.946–0.956)
Liu et al.^36^	Hand, foot, and mouth disease (HFMD)	to identify children at high risk of severe HFMD at hospital admission	2,532 children (mean age 24 months)	Rrandom forest, SVM, XGBoost, logistic regression, MLP	the random forest-based system achieved 0.824 sensitivity, 0.931 specificity, 0.916 accuracy, and an AUC of 0.916 in external testing
Masino et al.^22^	Infant sepsis	to develop EHR-based models for early recognition of infant sepsis	618 infants (median age 17 days)	eight ML classifiers, including logistic regression, SVM, Random forest, AdaBoost, GBM	six models achieved comparable performance with mean AUCs between 0.80 and 0.82
Lin et al.^37^	Myopia	to develop ML models to predict the onset of high myopia using big data	132,457 participants	random forest	the model demonstrated that machine learning can effectively translate large-scale data into clinically relevant predictions
Alpern et al.^23^	Pediatric sepsis	to estimate the probability of developing sepsis within 48 h	1,604,422 children aged 2 months-18 years	ridge regression, gradient tree boosting	models achieved AUCs of 0.92 for logistic regression and 0.94 for gradient boosting
Daniel et al.^27^	Type 1 diabetes	to assess whether machine learning could enable earlier detection of type 1 diabetes in primary care	2,445,730 children <15 years	SuperLearner ensemble, logistic regression	the predictive algorithm could substantially reduce the proportion of patients presenting with diabetic ketoacidosis at diagnosis
Herr et al.^35^	Rare genetic diseases	to assess EHR and NLP-based methods for identifying rare genetic diseases	659,139 pediatric patients (mean age 10.6 years)	rule-based NLP, ML-based NLP	rule-based NLP achieved higher precision (97.5%) than ML-based methods (73.5%); chart review confirmed diagnoses in over 90% of cases
Su et al.^28^	Suicide	to develop machine learning models to predict suicidal behavior among children and adolescents	41,721 patients aged 10–18 years	L1-regularized logistic regression	models achieved AUCs ranging from 0.81 to 0.86 across prediction windows, identifying 53–62% of suicide-positive cases at 90% specificity
Lin et al.^34^	*Staphylococcus aureus* infection	to predict the risk of community-onset *S. aureus* infection in children	22,366 children <19 years	maximum entropy models	the MaxEnt models achieved AUCs ranging from 0.769 to 0.839 in testing datasets

ASD, autism spectrum disorder; ML, machine learning; SVM, support vector machine; SVM (RBF), SVM with a radial basis function kernel; NLP, natural language processing; GBM, gradient boosting machine; AUC, area under curve.

However, existing literature has largely focused on predicting specific events, such as suicide, self-harm, and first-episode psychosis, rather than providing continuous predictions for the broader spectrum of psychiatric crises that may require urgent care or hospitalization. Looking ahead, the underlying assumption is that historical patterns can predict future psychological health crises, and that such patterns can be identified within real-world EHR data, despite its inherent sparseness, noise, errors, and systematic bias.^20^^,^^38^^,^^39^

Aside from the challenges mentioned above regarding the diagnosis of mental disorders in children, it is important to note that pediatric diagnoses present unique challenges, primarily due to the developmental and communicative limitations of very young or non-verbal children. These challenges are further exacerbated by the fact that many pediatric illnesses share overlapping symptoms, making it difficult to distinguish between conditions.^40^ Additionally, many common childhood illnesses exhibit similar signs, and several pediatric disorders are relatively rare.^16^ Currently, DL has emerged as the preferred ML approach for various machine perception tasks and has demonstrated significant utility in natural language processing, sequence prediction, and mixed modality data settings.^5^^,^^41^ In the medical domain, DL techniques have the potential to leverage the entire EHR, including free-text notes, to generate predictions for a wide range of clinical problems and outcomes.^42^^,^^43^^,^^44^ A previous study reported that a support vector machine using pupillometry from 50 children aged 10–12 years detected ADHD with a sensitivity of 0.773 and a specificity of 0.753 under nested 10-fold cross-validation.^45^ In another study, a DL model trained on neonatal brain MRI white-matter connectomes classified 84% of infants as above or below the median cognitive score at two years in an independent test set.^46^ Choi et al., in their retrospective multicenter study, found that the AI model significantly improved the performance of inexperienced radiologists and emergency physicians in diagnosing pediatric skull fractures on plain radiographs.^47^

Taken together, the diagnostic literature in pediatrics is uneven in maturity across tasks. The strongest evidence is seen in image- and signal-rich applications, such as radiology, digital pathology, MRI, and electroencephalogram (EEG), where the inputs are relatively standardized and the reference labels are usually better defined. By contrast, AI for behavioral and neurodevelopmental assessment still depends more heavily on small cohorts, variable phenotyping, and context-sensitive clinical judgment. In the near term, these tools are most convincing as aids to triage and interpretation, rather than replacements for specialist evaluation.^48^ Studies have shown that AI-assisted analysis of brain MRI can quantify myelination, detect subtle cortical malformations, and estimate regional and global brain volumes in suspected atrophy or hydrocephalus, which reduces inter-observer variability and provides reproducible metrics.^40^^,^^49^ Overall, AI systems are helping address some of these gaps by improving accuracy and efficiency.

### Prediction, prognostication, and risk stratification

**Figure 1:**
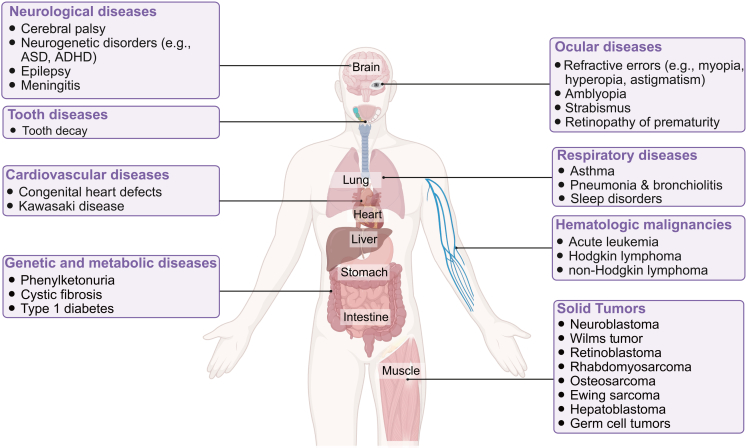
Clinical applications of artificial intelligence in the diagnosis and treatment of common pediatric diseases ASD, autism spectrum disorder; ADHD, attention-deficit/hyperactivity disorder.

AI has shown particular promise in pediatric risk stratification and prognostication, especially when multimodal clinical data are used to identify children at risk of future deterioration or adverse outcomes before those events become clinically evident.^7^^,^^40^ By leveraging multimodal data from EHRs, which include vital signs, laboratory results, medication histories, and clinical notes, AI models are able to identify children who are at risk for adverse health events before they become clinically evident. Meanwhile, this early identification is crucial, as it allows for timely interventions that can significantly improve a child’s long-term health prospects.^50^ Numerous studies have reported that AI shows great promise in predicting a wide range of pediatric conditions (Figure 1), including neurological diseases,^51^ psychiatric disorders,^52^^,^^53^ dental problems,^54^ ocular diseases,^55^^,^^56^ respiratory diseases,^57^^,^^58^^,^^59^ cardiovascular diseases, hematologic malignancies,^60^ acute kidney injury,^61^ sepsis,^23^^,^^62^ genetic and metabolic disease,^63^^,^^64^ and rare diseases.^65^^,^^66^^,^^67^ These advancements provide a scalable and adaptable framework for risk stratification, allowing pediatricians to not only identify at-risk children but also to implement preventive care strategies and timely intervention.

In clinical practice, early diagnosis and prediction are crucial for implementing timely strategies that can significantly improve patient outcomes. Taking pediatric intensive care units as an example, early warning systems that analyze real-time physiological data are being developed to detect subtle changes that may precede septic shock, respiratory failure, or cardiac arrest by several hours. This creates a critical window for timely intervention prior to the more serious manifestation of diseases.^68^^,^^69^ A recent study by Moeller et al. showed that the U-Sleep AI model effectively identifies sleep stages from normal PSGs in pediatric patients, performing at a level comparable to independent sleep experts.^59^ In other words, U-Sleep can streamline healthcare processes by reducing interrater variability and saving time, ultimately improving the evaluation of sleep disturbances. AI also advances prognostication and treatment response, offering new avenues for personalized treatment strategies.^70^^,^^71^ For children with complex chronic conditions such as cancer or autoimmune disease, anticipating individual trajectories and treatment responses is central to personalized care.^72^^,^^73^ Models that integrate clinical variables, molecular features, and treatment history can estimate survival, relapse risk, and the likelihood of long-term toxicities from therapies including chemotherapy and radiation.^70^ A systematic meta-analysis of 385 studies evaluated short-, mid-, and long-term survival and a range of adverse events. Across outcomes and subgroups, genomic-transcriptomic and AI-based models consistently showed superior predictive performance.^71^ In pediatric oncology, algorithms that combine histopathology with genomic markers can refine risk stratification beyond conventional staging.^74^ This may help spare low-risk patients unnecessary toxicity while focusing intensive treatment on those with high-risk features, and it supports informed shared decision-making with families.

Despite many encouraging performance reports, most pediatric prediction studies are still retrospective and developed within single institutions or health systems. Discrimination metrics such as AUC are often emphasized, but much less attention is given to calibration, subgroup performance, or whether these models change clinical decisions and improve outcomes when used in practice. Performance may also decline when models are transferred across sites, age groups, or workflows. For now, the most realistic role of predictive AI is to support monitoring, escalation, and risk stratification alongside clinician judgment, rather than to function as a stand-alone decision maker.

### Prevention and public health intervention

Similar to predictive characteristics, prevention is also an important aspect of child public health. Since the onset of the COVID-19 pandemic, neurodevelopmental issues among youth have risen substantially worldwide. Accurately predicting which children and adolescents in the general population will develop psychiatric problems would enable the efficient allocation of preventive resources.^75^ In the field of oral health, AI has been routinely used in pediatric dentistry to assist dentists in making more precise diagnoses, developing preventive strategies, and tailoring personalized treatment plans.^76^ Additionally, analyses of surveillance data can anticipate outbreaks of childhood infections, such as influenza or respiratory syncytial virus, enabling public health teams to mobilize vaccines and resources more effectively.^77^^,^^78^ Moreover, when combined with data on environmental exposures, socioeconomic indicators, and patterns of healthcare utilization, these approaches can identify neighborhoods or groups at higher risk of childhood myopia, lead exposure, or other preventable health issues, thus guiding targeted public health interventions.^79^^,^^80^

Compared with diagnosis and prognostic modeling, the preventive literature is still at an earlier stage. Many studies are valuable for identifying which children, families, or communities may be at higher risk, but fewer have shown that AI-guided interventions help reduce disease incidence, improve uptake of preventive services, or narrow disparities over time. At present, the most plausible near-term role for AI in prevention is to support surveillance and resource targeting, with stronger implementation evidence still needed.

### AI in pediatric surgery: Perioperative decision support and generative assistants

**Figure 2:**
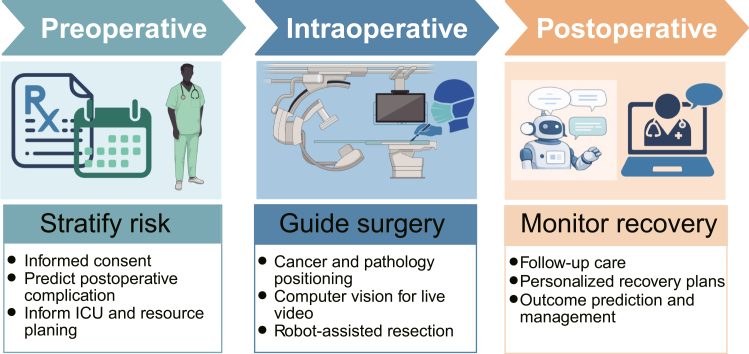
AI applications in pediatric surgery Risk stratification, image-based decision support, and postoperative monitoring for optimized surgical outcomes and recovery. ICU, intensive care unit.

AI in pediatric surgery is increasingly practical in perioperative risk stratification, image or pathology decision support, and workflow augmentation (Figure 2). In perioperative care, ML models can synthesize high-dimensional clinical and laboratory data to estimate complication risk and allocate resources more precisely than rule-based scores alone. For example, Tong et al. retrospectively studied 23,000 consecutive children (13,927 for training and 9,073 for testing) undergoing congenital heart surgery and compared five ML algorithms to predict major adverse postoperative outcomes (APOs). The established ML model can accurately predict the risk of four major APOs and the performance of ML models using only clinical variables was slightly lower than that using combined data.^81^

In pediatric surgical pathology, computer-vision models can reduce diagnostic delays for time-sensitive entities. Greenberg et al. developed an automated ganglion-cell detection workflow for Hirschsprung’s disease (HSCR) using 95 digital pathology slides for model construction and a validation cohort of 50 HSCR cases (727 slides). The algorithm identified ganglion cells with 96% sensitivity and 99% specificity (normal colon), enabled an expert to reach diagnoses using only algorithm-suggested regions with over 95% time savings, and reduced non-expert dependence on expert consultation to an estimated 20–58% of cases, depending on the case mix.^82^

Parallel to predictive and vision AI, LLMs are being explored as “generative assistants” in pediatric surgery, particularly for communication and documentation tasks. Recent pediatric-surgery studies highlight potential roles in drafting patient-facing materials and supporting clinician workflows, while emphasizing limitations, such as hallucinations, source opacity, and the need for governance, privacy safeguards, and guardian-centered consent processes.^83^^,^^84^^,^^85^^,^^86^ A key near-term opportunity is improving the readability and completeness of consent and discharge information. Azevedo et al. compared surgeon-generated informed consent documents with those generated by GPT-4o (AI-IC), collecting 102 paired responses from pediatric surgical patients. Legal representatives rated AI-IC higher across all quality parameters (*p* < 0.05), and pediatric surgeons scored AI-IC higher on most parameters, except conciseness (*p* = 0.06). Overall, 82.6% of legal representatives and 87.5% of pediatric surgeons preferred AI-generated consent documents.^87^ AI can enhance satisfaction with the informed consent process and potentially improve its quality in pediatric care. However, in pediatrics, these tools must also be evaluated in light of guardian-centered consent, child assent where appropriate, and the risk that automatically generated language may oversimplify uncertainty in high-stakes decisions.

However, it is important to note that diagnostic autonomy remains unsafe without strong guardrails. In a diagnostic study using 100 publicly available pediatric case reports, Barile et al. found the chatbot had a diagnostic error rate of 83% (83/100), and the final case-report diagnosis appeared in the LLM-generated differential list in only 36% (36/100) of cases, underscoring the risk of overtrust when LLMs are used for primary diagnosis.^88^ Consistent with broader concerns raised in previous commentary, LLMs may enhance care delivery but require tailoring to clinical needs and rigorous evaluation before high-stakes deployment.^83^^,^^85^^,^^89^

Overall, pediatric surgical AI appears closest to practical use in focused tasks such as perioperative risk estimation, pathology or image review, and communication or documentation support. Even so, much of the evidence remains retrospective or early-stage, and the safety threshold is especially high because errors can directly affect operative decisions, consent, and postoperative care. The contrast between promising workflow support and poor autonomous diagnostic performance is particularly important in this setting. In the future, these systems should be framed as supervised clinical assistants, not independent decision makers.

### Drug discovery and development

**Figure 3:**
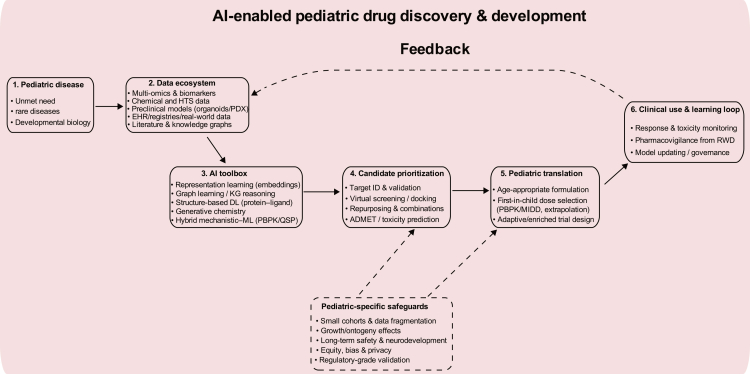
AI across pediatric drug discovery and development Schematic illustrating how multimodal pediatric data (biological, chemical, preclinical, and clinical real-world data) feed AI methods to support target and biomarker discovery, combination prioritization, dose selection via model-informed strategies (such as PBPK), trial enrichment/adaptive designs, and post-marketing pharmacovigilance. Pediatric-specific safeguards (developmental PK/PD, small cohorts, long-term safety, equity, privacy, and regulatory-grade validation) influence decision points throughout the pipeline and support a learning loop from real-world outcomes back to model refinement. ADMET, absorption-distribution-metabolism-excretion-toxicity; EHR, electronic health record; GNN, graph neural network; HTS, high-throughput screening; KG, knowledge graph; MIDD, model-informed drug development; PBPK, physiologically based pharmacokinetic; QSP, quantitative systems pharmacology; PDX, patient-derived xenograft; RWD, real-world data.

Pediatric drug discovery and development continue to lag behind adult indications because of small eligible populations, developmental heterogeneity in pharmacokinetics and pharmacodynamics, ethical constraints on sampling and trials, and limited commercial incentives. Consequently, off-label and unlicensed prescribing remains common in children, and the supporting evidence base may be limited.^90^ Many drugs prescribed for children are utilized off-label, often lacking sufficient age-appropriate evidence that can adequately inform dosing, efficacy, and safety.^91^ AI is a promising paradigm shift toward precision medicine in childhood cancer treatment, this include selecting the most effective chemotherapy drugs, identifying potential side effects, and adjusting treatment plans based on real-time patient responses (Figure 3). Multiple studies have illuminated the landscape of drug development in pediatric oncology, showcasing how AI tools can facilitate the identification of new drug combinations for the treatment of childhood cancers.^91^ For example, Carvalho et al. demonstrated that an AI-augmented approach significantly contributed to the identification of a novel drug combination aimed at treating diffuse intrinsic pontine glioma (DIPG) in children harboring ACVR1 gene mutations.^92^ Specifically, by pairing the drugs vandetanib and everolimus, the research team was able to enhance the permeability of vandetanib across the blood-brain barrier, thus opening up new avenues for the treatment of this previously incurable form of brain cancer.

Nowadays, studies show that AI and ML offer pragmatic opportunities to accelerate pediatric therapeutics by integrating heterogeneous data sources, including omics, chemical screens and real-world clinical data, and supporting decision-making across the discovery-development continuum. Across the pharmaceutical pipeline, ML approaches have been applied to target identification, biomarker discovery, lead optimization, ADMET prediction, and trial enrichment; however, clinical and regulatory translation depends on data quality, transparent evaluation, and prospective validation.^93^^,^^94^ In addition, ML-enhanced physiologically based pharmacokinetic (PBPK) modeling can simulate how drugs are absorbed, distributed, metabolized, and excreted in children’s bodies, which vary significantly with age, weight, and body composition.^95^^,^^96^^,^^97^ These silico simulations can predict optimal dosing regimens for pediatric populations, minimizing the need for extensive and invasive experimental studies. Additionally, AI is also accelerating drug repurposing by analyzing vast biomedical databases containing molecular structures, biological pathways, and drug effects.^91^^,^^98^ This enables the identification of existing drugs approved for adult conditions that might be effective against rare pediatric diseases, offering a faster and more cost-effective alternative to developing new drugs from scratch.

AI also plays a crucial role in discovering novel therapeutics, particularly in the realm of targeted treatments for pediatric cancers that are often driven by specific genetic alterations.^99^^,^^100^ DL models can analyze the intricate structure of proteins and predict how potential drug molecules might interact with them.^97^^,^^101^ This approach moves away from non-specific cytotoxic agents, allowing for precision medicine with fewer side effects.^91^^,^^102^ In summary, AI is currently contributing most clearly upstream in the pipeline, including target discovery, drug repurposing, virtual screening, and pediatric PK/PD modeling. However, much of the evidence remains preclinical, *in silico*, or based on heterogeneous datasets that are difficult to standardize across pediatric age groups and disease settings. Regarding translation into pediatric trials, it is worth noting that dosing decisions and regulatory practice remain limited, in part because developmental biology and small patient populations make validation especially challenging.

### Global child health and resource-constrained settings

AI has significant potential to improve child health in both low- and middle-income countries (LMICs) as well as high-income countries (HICs).^103^^,^^104^^,^^105^ While many advancements have been primarily directed toward the needs of HICs due to their greater healthcare resources, AI technologies can effectively address the challenges faced by health systems in LMICs, where shortages of healthcare workers and resource constraints are prevalent. Taking vaccination as an example, AI algorithms can analyze large immunization datasets to identify trends and underserved areas, providing critical insights into vaccination coverage and clinical practices affecting children’s health. Numerous studies indicate that mobile applications designed to provide vaccine-related information can significantly improve vaccination knowledge among parents.^106^^,^^107^ These applications have shown promise in boosting users' understanding of vaccination schedules and history, receiving positive feedback for their reminder features for booster shots and overall tracking of children’s vaccination status. Furthermore, AI tools can help community health workers diagnose conditions such as malaria or detect early signs of skin cancer using simple imaging techniques. As smartphones become more accessible, these tools can also guide lifestyle choices and offer support during pregnancy and recovery.^103^^,^^108^

Another significant way to leverage AI in child health is by developing low-cost, AI-powered tools designed for non-specialist health workers. These tools can greatly enhance diagnostic capabilities and provide immediate recommendations, especially in resource-limited settings. Nowadays, AI-assisted diagnostic tools can analyze medical images such as X-rays, CT, ultrasounds, and skin photographs to identify common diseases, including pneumonia, malaria, or skin cancer.^109^^,^^110^ These AI systems assist non-specialist health workers in accurately diagnosing conditions that might otherwise be missed, helping to improve diagnostic efficacy and improve treatment outcomes.^104^^,^^108^^,^^111^ In general, the promise of AI for global child health is substantial, especially in settings where specialist access is limited, but generalizability across contexts remains a major concern.

## Future perspectives and challenges

**Figure 4:**
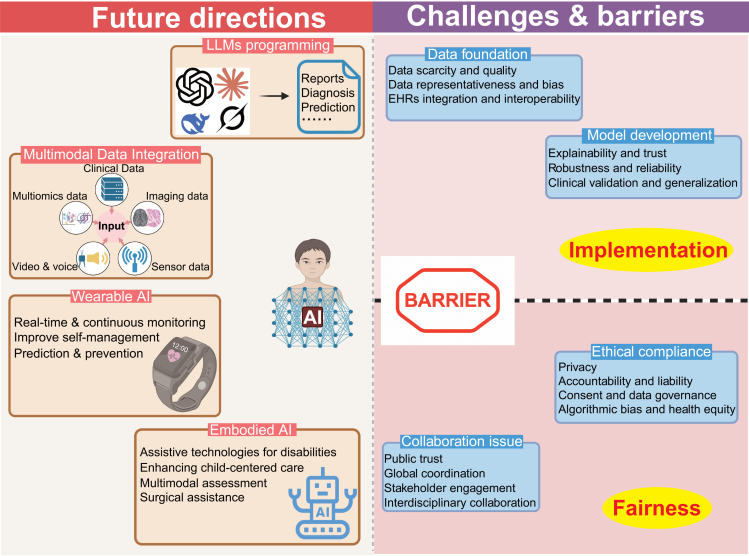
Future directions and key challenges in implementing artificial intelligence (AI) for children’s health LLMs, large language models.

The future of AI in pediatric health holds immense promise, as emerging technologies continue to push the boundaries of data integration and personalized care. Advanced neural networks capable of integrating multimodal data, ranging from imaging and genomics to continuous wearable sensor data, are poised to transform how pediatric diseases are diagnosed and managed.^112^ By creating comprehensive biomarker profiles, AI could offer earlier, more accurate diagnoses and guide personalized interventions, particularly for complex conditions such as neurodevelopmental disorders. However, as AI advances, several challenges must be addressed. Ethical considerations around data privacy, bias, and transparency remain critical, especially when dealing with vulnerable pediatric populations. Moreover, overcoming practical barriers such as incompatible data systems and regulatory challenges will be essential for AI’s widespread adoption (Figure 4).

### Advanced data-driven approaches for multimodal data integration

The frontier of AI in child health is being pushed forward by advancements in foundational models and their application to novel data types, including multi-omics data (e.g., genome, epigenome, proteome, transcriptome, and metabolome) and imaging.^113^^,^^114^ These technologies move beyond assisting discrete clinical tasks and toward building a holistic, integrated understanding of a child’s health and development. The future of pediatric AI lies not in analyzing data in isolation, but in the synergistic integration of diverse data modalities. DL architectures are now capable of fusing imaging, genomics, proteomics, metabolomics, and continuous data from wearable sensors. For a child with a neurodevelopmental disorder such as ASD, an AI model could theoretically integrate structural MRI data, electroencephalography patterns, genetic sequencing results, and behavioral data coded from video recordings to create a comprehensive biomarker profile.^115^^,^^116^ This could lead to earlier and more accurate diagnosis, predict which children are likely to have co-morbid intellectual disability or epilepsy, and suggest which behavioral or pharmacological interventions might be most effective for a particular child’s biological subtype.

In addition, the application of LLMs such as generative pretrained transformers (GPTs) in medicine is nascent but holds extraordinary promise for child health, particularly in the domains of development and psychiatric conditions.^117^^,^^118^ LLMs can be fine-tuned on vast corpora of pediatric medical literature, guidelines, and de-identified clinical notes to become powerful specialized assistants. In clinical settings, LLMs can alleviate administrative burdens by transcribing doctor-patient conversations into clinical notes and generating tailored discharge summaries. They can also enhance health literacy by translating complex medical terminology into age-appropriate language and even suggesting visual aids, such as illustrations or cartoons, to engage and educate younger patients. Beyond documentation and education, LLMs and related natural language processing tools can support objective developmental and mental health screening. By analyzing a child’s speech patterns, vocabulary diversity, and syntactic complexity during natural interactions, these models can help flag potential language delays, dyslexia risk, or early signs consistent with ASD.^119^^,^^120^ We are concerned, however, that such applications also raise pediatric-specific concerns because early algorithmic labeling in developmental or mental health contexts may influence family perceptions, clinical expectations, and access to services if not communicated and governed carefully.

### The integration of AI and wearable technology

The rise of wearable devices, augmented by the capabilities of AI, has revolutionized the healthcare landscape by enabling continuous monitoring and data collection on various physiological and behavioral metrics. The fusion of AI and wearable technologies presents promising opportunities for early diagnosis and personalized management of chronic conditions, particularly in pediatric populations. A randomized clinical trial indicated that a wearable social learning aid significantly improved the social skills of children with ASD between the ages of 6 and 12 years as an augmentation to standard of care therapy.^121^ Moreover, wearable technologies have been successfully implemented for managing other chronic conditions such as type 1 diabetes, cerebral palsy, epilepsy and obstructive sleep apnoea, showcasing their versatility in diverse health scenarios.^122^^,^^123^^,^^124^ As healthcare systems increasingly adopt AI-integrated wearables, they usher in a new paradigm of proactive health management that could transform clinical practice.

### Application of embodied AI in pediatrics

Embodied artificial intelligence (EAI) is an emerging paradigm that integrates AI into physical agents such as robots, enabling perception, learning, and action through continuous interaction with the environment.^125^ By unifying perception, memory, reasoning, and action, EAI supports multimodal, dynamic learning with real-time feedback. Unlike conventional models trained on static datasets, EAI systems process online multimodal inputs for context-aware adaptation to changing conditions, allowing them to tackle complex, real-world problems. Recent advances in LLMs, vision-language models, and reinforcement learning have accelerated this progress and provided an intelligent control core suited to clinical tasks that require personalization and responsiveness.^126^ In pediatrics, EAI shows promise across clinical domains, including disease screening and diagnosis, perioperative and surgical assistance, assistive technologies for children with visual or motor impairments, medical education and simulation, and data-driven clinical research. As the field matures, we believe that EAI will complement existing medical AI with a stronger emphasis on safety, human oversight, and child-centered care.

### Challenges and barriers

Integrating AI into pediatric medicine, while promising, is fraught with significant challenges (Box 1).^1^ Firstly, data scarcity and representativeness remain fundamental barriers. Ethical safeguards rightly restrict the collection of children’s health data, which leads to small and uneven datasets.^15^^,^^118^ Age bands, developmental stages, ethnicity, and socioeconomic status are often underrepresented. For embodied systems, the lack of large, real-world interactive data further limits robust learning and generalization across settings and devices. Secondly, transparency is imperative. Healthcare providers need to comprehend the reasoning behind AI’s conclusions when making clinical decisions, particularly when children’s well-being is at stake. The “black box” nature of many AI models complicates this, as decision-making processes are often obscured, creating an additional layer of risk in pediatric applications.^113^ Many deep models function as black boxes, which hampers clinical trust and error analysis. Specifically, in high-stakes fields such as pediatric oncology, where diseases are often driven by genetic abnormalities and treatments can be highly toxic, clinicians need clear rationale, calibrated uncertainty, and prospective validation. Thirdly, regulatory hurdles complicate implementation. There is an absence of established protocols governing the deployment of AI in pediatric healthcare, which further obstructs its integration into clinical practice. Incompatibility among data systems and existing healthcare frameworks adds to these challenges, limiting the scalability of AI solutions.

Box 1 **Pediatric governance, bias, and fairness considerations for AI in child health**

AI governance in pediatrics must account for risks that are distinct from adult medicine because children are developing biologically, cognitively, and socially, and because data collected early in life may influence care over many years.

**Developmental heterogeneity matters.** Pediatric populations should not be modeled as a single age group. Model performance, calibration, and error profiles should be reported across clinically meaningful age bands, developmental stages, and disease subtypes, with reassessment over time as children grow and care contexts change.

**Consent is family-centered but evolves over time.** Because minors cannot usually provide full legal consent, pediatric AI systems require careful attention to parental permission, age-appropriate assent where feasible, and transparent communication regarding data use, model limitations, and downstream clinical implications.

**Digital footprints begin early and may persist.** Training and evaluation datasets should reflect variation in sex, ethnicity, language, disability, comorbidity burden, geography, and socioeconomic context. Aggregate performance metrics are insufficient; subgroup performance, missing-data patterns, and calibration should be reported directly.

**Longitudinal digital footprints raise distinctive privacy risks.** Pediatric data may accumulate from infancy across EHRs, imaging, genomics, wearables, and other linked systems. Data minimization, controlled linkage, secure governance, and clear retention policies are particularly important because re-identification or downstream misuse may have long-term consequences for children and families.

**Fairness must be demonstrated, not assumed.** Aggregate model performance is insufficient. Subgroup reporting should include age, sex, ethnicity, language, disability, geography, and socioeconomic context where feasible.

**Predictive labeling can cause harm.** Risk models in areas such as mental health, neurodevelopment, obesity, or adherence can influence clinician expectations, family decision-making, and access to services. Such outputs should be used to support timely and equitable care, not to stigmatize children or justify punitive responses.

**Human oversight remains essential.** High-stakes pediatric AI tools should be deployed with clearly defined clinical responsibility, override mechanisms, audit trails, and pathways for error review. Good governance also requires prospective evaluation, periodic recalibration, drift monitoring, and assessment of unintended consequences on equity, workflow, and patient outcomes.

A further challenge is the gap between model performance and deployment evidence. High AUC values alone are insufficient for clinical adoption. Pediatric AI systems should ideally demonstrate calibration, subgroup fairness, external validation across age strata and care settings, human-factors evaluation, and prospective evidence of workflow or patient benefit. This evidence hierarchy is especially important in pediatrics because developmental change makes model drift and performance decay more likely over time. Moreover, reproducibility in pediatric AI is further limited by inconsistent public release of code and software pipelines, and by the fact that relatively few studies have resulted in stable, publicly accessible tools and web platforms, making independent validation and comparison across studies difficult. Finally, effective collaboration among healthcare professionals, researchers, and policymakers is essential to overcome these obstacles. To fully realize AI’s potential in improving pediatric diagnosis and treatment, it is crucial to cultivate an environment conducive to innovation that addresses ethical, technical, and regulatory challenges.

## Conclusion

Overall, pediatric AI is advancing rapidly, but the field remains uneven in maturity. Image-based assistive tools, structured risk prediction models, and clinician-facing documentation support appear closest to cautious implementation, whereas autonomous diagnostic or treatment-recommendation systems still require substantially stronger pediatric-specific evidence, fairness auditing, and governance before routine use. Achieving these prerequisites will depend on sustained collaboration among AI researchers, pediatricians, ethicists, and regulators to ensure that tools are technically robust, ethically sound, and aligned with children’s developmental needs. With the right safeguards in place, AI can pave the way for a new era in pediatric healthcare, where the focus is on timely, accurate diagnoses and improved clinical outcomes, ultimately leading to healthier futures for children worldwide.

## Acknowledgments

This study was supported by the 10.13039/501100001809National Natural Science Foundation of China (no. 82403017), General Research Fund from RGC (no. 14122725), the RGC Postdoctoral Fellowship Scheme (PDFS, RGC Reference No. UGC/GEN/562/3). Graphical abstract and Figures were created in BioRender.

## Author contributions

W.K. conceptualized the study and provided direction and guidance on the whole project. J.L.W. and B.N.C. wrote and edited the manuscript. Y.L., P.Y.Y., T.J.F., and F.D.X. performed the literature search and revised the manuscript. K.C.-C.C., M.Y.L., S.F.Y., F.B.Z., and T.C.F.Y. gave professional comments on the study. All authors reviewed and approved the final version of the manuscript.

## Declaration of interests

T.C.F.Y. has served as an advisory committee member and a speaker for Gilead Sciences. The other authors declare that they have no competing interests.
